# A Networked Intelligent Elderly Care Model Based on Nursing Robots to Achieve Healthy Aging

**DOI:** 10.34133/research.0592

**Published:** 2025-01-22

**Authors:** Wenting Ji, Ruzhen Luo, Yumei Sun, Maiping Yang, Yueheng Liu, Hua Chen, Dongmei Lin, Ziyi Su, Guangming Tao, Diansheng Chen, Hongyu Sun

**Affiliations:** ^1^ Chengdu University of Traditional Chinese Medicine, Chengdu 611137, China.; ^2^ School of Nursing, Tianjin Medical University, Tianjin 300070, China.; ^3^ Peking University, Beijing 100191, China.; ^4^ Wuhan National Laboratory for Optoelectronics, Huazhong University of Science and Technology, Wuhan 430074, China.; ^5^ School of Nursing, Gannan Medical University, Ganzhou 341000, China.; ^6^ Key Laboratory of Vascular Aging, Ministry of Education, Tongji Hospital, Tongji Medical College, Huazhong University of Science and Technology, Wuhan 430030, China.; ^7^ School of Materials Science and Engineering, Huazhong University of Science and Technology, Wuhan 430074, China.; ^8^ School of Physical Education, Huazhong University of Science and Technology, Wuhan 430074, China.; ^9^ Research Center for Intelligent Fiber Devices and Equipments and State Key Laboratory of New Textile Materials and Advanced Processing Technologies, Huazhong University of Science and Technology, Wuhan 430074, China.; ^10^ Department of Geriatrics, Tongji Hospital of Tongji Medical College, Huazhong University of Science and Technology, Wuhan 430030, China.; ^11^ School of Mechanical Engineering and Automation, Beihang University, Beijing 100191, China.

## Abstract

As global populations become increasingly aged, existing elderly care models are proving insufficient. The development and application of nursing robots have shown potential in addressing the challenges of elder care in aging societies. This perspective outlines current state and potential applications of nursing robots in promoting healthy aging. Given this background, a networked intelligent elderly care model for nursing robots, which integrates technologies such as big data, artificial intelligence, the Internet of Things, and nursing robotics, is proposed. This model would synergistically combine elderly health monitoring, capability assessment, and intelligent allocation functions to revolutionize global elderly care practices and promote healthy aging.

## Introduction

The proportion of the global population aged 65 years and above is increasing, with an expected increase from 10% to 16% by 2050 [1]. For elderly people, the care needs of an individual become more complex and extensive; indeed, a survey of 24,000 community-dwelling elderly people revealed that 8.52% of them face difficulties in daily activities such as eating, dressing, and bathing [2], many of which require strong support from nursing staff.

Nursing care involves a range of entities, from hospitals to communities and families, and encompasses fields such as geriatric care, chronic disease management, and rehabilitation guidance [3]. However, geriatric nursing demands high professionalism and workloads and involves challenges related to unmet long-term care needs, a shortage of human resources, and major financial burdens on the patients’ families and society [4–6]. This situation has formed the elderly care crisis. Enhancing the technological level and integration of elderly health services and actively addressing aging are essential strategies for all countries.

With advancements in artificial intelligence, the Internet of Things (IoT), and other digital technologies, elderly care services now incorporate concepts, such as precision care, proactive health, and lifelong comprehensive care, to transform the aging paradigm. Critically, the concept of active health proposed in the "Healthy China 2030" plan emphasizes the creation of health value through preventive measures and the management of health risk factors at their source [7]. Specifically, the traditional elderly care should be transformed into an integrated medical and health services model that combines nonpharmacological and pharmacological interventions for prevention, diagnosis, treatment, rehabilitation, and daily care [8]. Technological advancements like nursing robots, remote medical interventions, mobile health applications, and remote monitoring devices are becoming increasingly common in communities and households, addressing challenges related to healthcare accessibility, availability, and cost [9,10]. Examples include remote check-in systems [11], metaverse care platforms [12], and IoT smart home environments [13]. Among these technologies, nursing robots are crucial for addressing the elder care crisis [14], transforming traditional nursing models, and creating innovative nursing approaches.

Nursing robots are partially or fully autonomous machines that provide care-related services to individuals with physical or intellectual disabilities [15]. They can offer physical, cognitive, and emotional support, addressing challenges in the users’ daily lives [16]. Advancements in several cutting-edge fields, including embodied intelligence and vertical macromodeling [17], bionic robotics [18], 3D perception modeling and multimodal information fusion [19], and robotic operating systems [20], have enabled the widespread application of nursing robots, providing various solutions to improve the health management and quality of life [21]. Nursing robots are typically used in health risk monitoring, assisting with daily activities, functional exercise, and social interaction, providing companionship and support for elderly people [22–25]. Depending on their functionality, nursing robots come in various forms, including humanoid designs, animal-like forms, and specialized functional shapes. Examples include the humanoid robot Pepper, the seal-shaped robot Paro [26], and the multifunctional robotic arm Lio, which are already used in homes and care facilities worldwide [27]. Compared to human care, robot-assisted care offers greater predictability and transparency and allows the patient to regain independence in daily living. Furthermore, effective interaction with nursing robots can address elderly people’s psychological and physiological needs [28].

Given the scarcity of resources, economic burdens, and cultural perspectives, community-based home care is becoming the mainstream global elderly care model. For example, in Japan, 24 million elderly people receive care at home [29], while in China, a family- and community-centered elderly care model has emerged, in which 90% of elderly people receive home-based care and 7% receive community-based care [30]. Similarly, in the United States, laws [31] and institutions [32] have been established to provide services for community-dwelling elderly people, enabling them to live independently at home. However, elderly individuals receiving home care often have unmet needs related to health management, home rehabilitation training, daily living assistance, and emotional support [33,34]. Nursing robots can help address these needs by partially filling the gaps in traditional home-based elderly care models.

Firstly, this article comprehensively reviews and analyzes the potential of nursing robotics in addressing the global aging crisis. The latest technology in nursing robots, their clinical applications—including health monitoring, functional exercise, daily activity assistance, and social support—and future research and developmental directions are discussed. The review also addresses the limitations and challenges of current nursing robotics technology, including intelligence bottlenecks, data privacy concerns, and ethical considerations. Then, a networked intelligent nursing model is proposed to better utilize the capabilities of nursing robots. This model would establish a networked intelligent elderly care system that optimizes the configuration of nursing robots and combines their capabilities in health status monitoring, classification of activities of daily living, and comprehensive physical, psychological, and social assessment. The model would be built on the basis of 5 dimensions: (a) networked collaboration, (b) intelligence-driven services, (c) precision service delivery, (d) diversified integration, and (e) standardized development. Implementing such a networked intelligent nursing model can help unlock the full potential of nursing robots and provide a new and feasible path for achieving global health.

## The Use of Nursing Robots for Achieving Healthy Aging

**Fig. 1.**
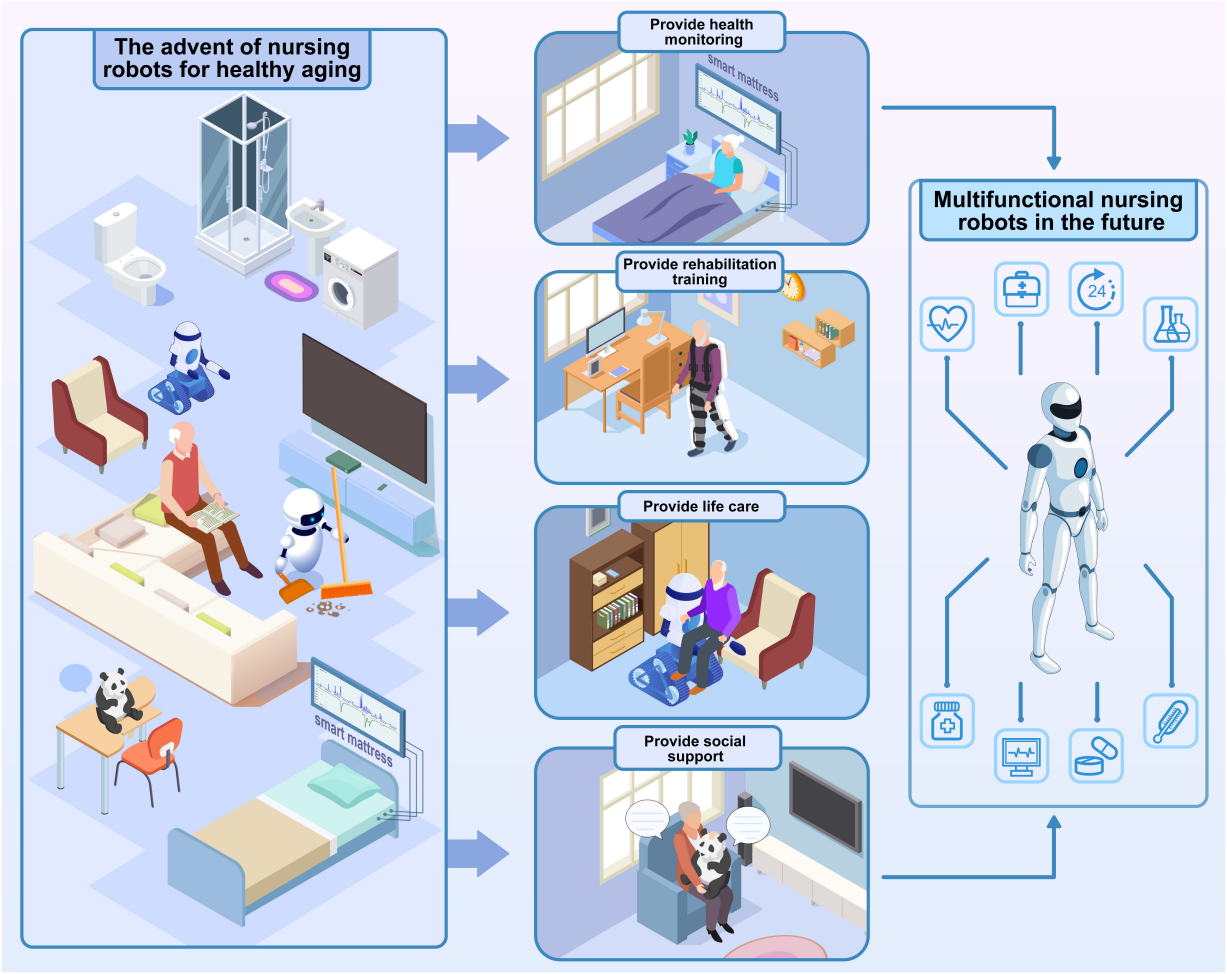
Nursing robot assistance in healthy aging.

Currently, nursing robots can provide health monitoring, rehabilitation training, life care, and social support separately. Multifunctional nursing robots in the future would be popular in addressing elderly care crisis in place of traditional home-based elderly care models (Fig. 1).

### Health monitoring

Home health monitoring is an emerging field in healthcare that is particularly suitable for frail elderly and chronic disease patients. Surveys have indicated that most elderly people prefer wearable technology for monitoring their health, especially when a diagnosed patient must be monitored and individuals who are specifically experiencing symptoms [35]. Health monitoring robots offer high controllability, repeatability, and precise measurement capabilities. For example, the RoBoHoN telephone robot, which is equipped with facial recognition technology, functions as a nighttime accident monitor [36]. In addition to the ability to make phone calls, it has dual-function eyes that can project images and capture video, and it automatically manages email communications to keep caregivers updated about any incidents. Z-Works, an intelligent sensor company, produces a 3-sensor system that monitors various aspects of the home environment. The first sensor, placed on the bed, measures heart rate, respiratory rate, the amount of ambient light, temperature, and movement. The second sensor acts as a motion detector by monitoring light, temperature, humidity, and movement. The third sensor is placed on the door and detects whether it is open or closed. In 2017, Z-Works secured $3.6 million in Series A funding and collaborated with Canon Marketing Japan to deploy remote monitoring systems in 115 nursing homes in Japan [37]. Mimamori Eye [38] is a location-based smartphone app that creates a secure online community for elderly people. Caregivers can register the physical descriptions and photos of elderly people in the app; subsequently, if elderly people go missing, the app will alert all users within a 20-km radius. Safety Net Linkage, the company behind this technology, also operates a dedicated hotline for reporting missing and found elderly people.

Artificial intelligence can enhance the monitoring of patients’ conditions, such as the appearance and conditions of any wounds, through machine vision technology, performing tasks beyond the capabilities of human vision. For example, Hurley et al. [39] used a real-time bacterial fluorescence imaging device to detect potentially harmful bacteria levels by capturing fluorescence images on the basis of endogenous autofluorescence. The red fluorescence of most gram-positive, gram-negative, aerobic, and anaerobic bacteria was detected in moderate to severe colonized wounds, whereas cyan fluorescence was observed only in the presence of *Pseudomonas aeruginosa*. Armstrong et al. [40] analyzed the role of fluorescence imaging in detecting biofilm-encased and planktonic bacteria in large-volume wounds with machine vision technology. The results revealed that 84.2% of the ulcers had a high bacterial load. In general, nursing robots collect vast amounts of real-world data during health monitoring, which, if fully utilized (e.g., for user profiling and disease prediction models), can provide more targeted care services for elderly people. Artificial intelligence is a powerful tool for processing these data from monitoring and alerting, bridging technological gaps, and optimizing resource allocation, effectively addressing the challenge of “unmanned work”. In the future, we expect that more artificial intelligence tools will be integrated in remote healthcare to expand home care systems.

### Rehabilitation training

Interest in the use of robots in rehabilitation therapy is growing because the ability of these devices is essential for physical therapy or upper and lower limb recovery. These robots can adapt rehabilitation protocols suitable to the stage of the patient’s progress [41]. Conducting rehabilitation training at home with robots offers several benefits, including more effective integration of training routines into daily life and reduced travel burdens and healthcare resource use [42]. Nursing robots are playing increasingly important roles in home-based functional exercise [43], such as by encouraging users to perform simple dance movements [44], assisting with walking training [45], or serving as an alternative for various fitness equipment for general exercise [46]. For example, the Rewalk system, approved by the U.S. Food and Drug Administration in 2014, is used worldwide to help patients with complete spinal cord injury walk up to 100 m during an average of 13 to 14 training sessions without adverse effects. The Multi-Stage Hemiplegic Lower Extremity Rehabilitation Robot [47] provides lower extremity motor rehabilitation for hemiplegic patients at different stages of recovery. It features multilevel availability, bilateral usability (i.e., for training the left or right limb), a compact design, adjustability, and portability. Nam et al. [48] proposed an electromyography-driven peripheral nerve muscle exoskeleton that integrates neuromuscular electrical stimulation (NMES), soft pneumatic muscles, and exoskeleton technology for autonomous upper limb training after stroke. The device also supports remote autonomous rehabilitation, allowing staff to monitor its status through logged data [49]. Taken together, this evidence suggests that nursing robots have potential in assisting with home-based rehabilitation exercises. Unsupervised robot-assisted rehabilitation is feasible and could complement routine clinical care, especially when applied in home settings [50].

### Life care

Mobility limitations [51] and fall risks [52] are important barriers to independent living for elderly people, who often lack the coordination and strength required to perform their daily activities. Nursing robots can address these concerns by effectively assisting elderly people with mobility challenges, such as performing basic motions, maintaining stability, and maintaining personal hygiene. Some nursing robots can be manipulated to or automatically pick up and deliver necessary items [53,54]. In terms of mobility assistance, ROBEAR [55], a care robot developed by the RIKEN Research Institute and Sumitomo Polytechnic Co. in Japan, was designed to assist elderly people in performing gentle movements, such as lifting or lowering themselves or standing up. The researchers also developed a multimodal intelligent wheelchair robot [56] that can effectively assist elderly individuals living alone in performing movements such as standing, thereby reducing the burden of physical activity. Additionally, for stabilization support, another device, the “Handle Anywhere” armrest robot, features an omnidirectional mobile base attached to a repositionable handrail, providing support for elderly individuals in achieving various positions and postures [57]. A support system for assisting with dressing has been designed, consisting of 2 robotic arms [58]: one arm places the shirt sleeve on one arm, whereas the other moves the arm to the appropriate dressing position. With respect to personal hygiene, elderly people have reported success and positive experiences in operating a robot to shave themselves independently [59].

By 2030, nearly 60 million severely disabled elderly people in China are expected to be in need of care. In frail elderly people [60], urinary or fecal incontinence is common, affecting the patient’s quality of life and increasing the caregivers’ burden [61,62]. EverCare [63] is a fully automated defecation system designed to address the most inconvenient and burdensome aspects of bowel care. Sensors in the system detect feces or urine within 2 s of discharge, then the system automatically collects the products and stores them in a waste bucket. It subsequently sprays clean water at an appropriate temperature through a nozzle to cleanse the private areas and the toilet bowl and applies heated air to dry the patient’s body. The system operates automatically around the clock, maintaining the dignity of the patient.

Although nursing robots cannot yet match the expertise of professional caregivers, they can perform many tasks to assist elderly people in living independently with dignity. While most designs are currently in the developmental phases or only implemented in small-scale scenarios, robot technology holds promise as an auxiliary tool for addressing current aging challenges and paving the way for healthy aging.

### Social interaction and emotional support

In addition to physical needs, the emotional and social needs of elderly people are also important for promoting healthy aging. Elderly individuals face various physiological and psychosocial challenges, such as declining physical function, loss of intimate relationships, and social isolation, all of which can lead to psychological health issues such as dementia, depression, and anxiety. Through scientific and technological innovations, social assistive robot interventions have been implemented in the field of elderly care [64]. These robots possess social skills and human/animal-like features powered by artificial intelligence [65], allowing them to interact with or exchange information with elderly people to meet psychological health needs [66]. Existing social robots serve various functions, including emotional therapy, cognitive training, social facilitation, companionship, and physiological treatment [67]. Studies have confirmed the effectiveness of social robots in providing psychological health care [68] and elderly-specific care [69], enhancing elderly people’s psychological well-being by providing high levels of emotional support and encouraging social interaction [70].

Social robots can also help elderly people establish and maintain social relationships. Scholars have suggested that the design of social robots should focus on 3 aspects to address the needs of elderly adults, namely, value aspects (interpersonal communication and emotional support), behavioral aspects (medical companionship and cognitive stimulation), and ontological aspects (intelligent recognition technology, multimodal technology, and augmented reality technology) [71]. First, social robots can provide emotional support to elderly people. By recognizing the emotions and behaviors of elderly people, robots can validate both positive emotions such as happiness and excitement and negative emotions such as anxiety and depression. Pet robots such as Peth [72] are equipped with tactile sensors and can recognize verbal and nonverbal emotions in elderly people. Similar robots include Paro, which is modeled on the appearance of a young harp seal and has functions mimicking 4 primary senses: sight, hearing, balance, and touch [25]. This social robot was developed for therapeutic purposes by Japan’s Advanced Institute of Science and Industrial Technology, inspired by living animal-assisted therapies to improve the physiological and emotional conditions of elderly inpatients and reduce agitation in dementia patients. A systematic review revealed that Paro may be a helpful nonpharmacological approach for improving behavioral and psychological symptoms, reducing medication use, and improving social competence in people with dementia [73].

Second, social robots can provide support at the behavioral level, such as by engaging in conversations and relaxing with elderly people. PaPeRo [74] communicates with elderly people with dementia through human–computer interaction and holds basic conversations to support independent living. The robots Ed [75] and MARIO [76] were designed with humanoid characteristics: The head component comprises a liquid crystal display screen display, whereas the body component incorporates a speaker, a camera, and a microphone for making calls, reading news, playing games that provide cognitive stimulation, and reminding the individual of his or her daily schedule.

Social robots can also cognitively stimulate elderly people in an attempt to slow cognitive decline. This can be achieved by capturing the patient’s attention, promoting movement and activity, activating cognition through communication, and assisting in memory recall. Audrey is a social robot designed to assist elderly people living alone, providing proactive reminders to reach out to family members and facilitating intergenerational communication [77]. Other systems with similar functions include dialog systems [78,79], family conversational robots [80], social robots with intellectual stimulation and gaming capabilities [77], and social robots that provide examples of participatory arts, such as theater. Social assistive robots have been demonstrated to positively impact elderly people’s mental health, with evidence suggesting that they facilitate communication, alleviate dysphoria, improve cognition, and reduce the burden on caregivers [81,82]. Nevertheless, there are opportunities to develop social robots further. Some scholars have proposed that information exchange and interaction between elderly people and the outside world could be enhanced by allowing pet robots to learn new skills [83] or achieve greater interactivity [84] and by designing more activities centered around the robots [85]. Čaić et al. [86] proposed that social robots should exhibit empathy, flexibility, and spontaneity, similar to those exhibited by human coaches.

### The future of nursing robots

As elderly care demands grow with the increasing aging population, the multifaceted challenges regarding chronic disease management, disability support, and resource allocation are becoming increasingly evident. Nursing robots, with their expanding functionalities, present a promising solution to these challenges. We have introduced how nursing robots can be applied in elderly care in terms of health monitoring, rehabilitation training, life care, and social support. In the future, nursing robots are expected to become integral elements of comprehensive care systems, tailored to address specific healthcare needs. Recent innovations have led to the development of multifunctional devices that implement a body transfer mechanism [87], assisting disabled elderly people in transferring between nursing beds, wheelchairs, and hygiene cabins. A team from Stanford developed “Aloha” [20], a robot capable of performing various household tasks and learning new functions to aid in daily living activities. Similarly, “Lio” by the Swiss F&P Robotics team [26], which is already operational in several European care facilities, offers a broad spectrum of services, from health monitoring to emotional companionship. Given the enormous potential demonstrated by these nursing robots, they are well positioned to address the challenges of aging. However, current nursing robot products typically offer limited functionality and lack unified production and usage standards. Moreover, these robots have yet to be fully integrated with technologies such as big data and cloud platforms, and thus their potential has not been fully unleashed. How can we incorporate nursing robots into existing elderly care models and efficiently resolve their problems?

## Building a Networked Intelligent Elderly Care Model Based on Nursing Robots

**Fig. 2.**
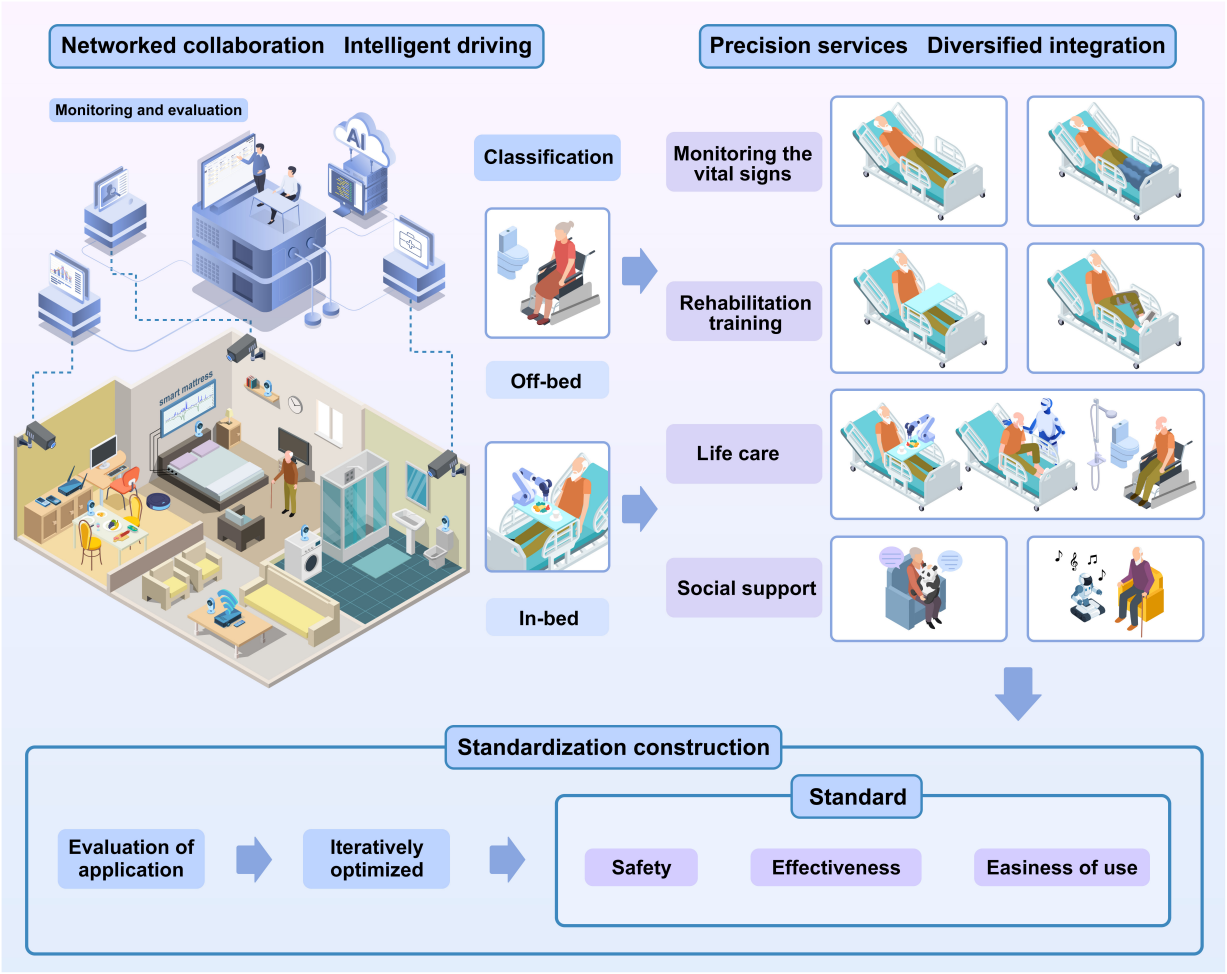
Building a networked intelligent elderly care model based on nursing robots.

Based on the development of nursing robots, we propose a networked intelligent elderly care model for nursing robots to promote healthy aging that will leverage a number of cutting-edge technologies, including artificial intelligence, cloud computing, and the IoT. This model would integrate health monitoring, classification of activities of daily living, comprehensive physical, psychological, and social assessment, and resource allocation, paving the way for transformative advancements in elderly care. The framework of this model would include networked collaboration, intelligence-driven services, precision service delivery, diversified integration, and standardized development (Fig. 2). These components would work together to create a robust system capable of providing personalized, responsive care in institutional, community, and home settings. Challenges such as data privacy, autonomy in decision-making, and the emotion-emulating capabilities of the robots would be addressed through continuous technological advancements and the establishment of appropriate regulations. Ultimately, the aim of the model is to offer a sustainable, effective solution to meet the complex needs of the elderly population, enhancing their quality of life and fostering independent living.

### Networked collaboration

Networked collaboration is essential for maximizing the efficiency and effectiveness of nursing robots in elderly care. This aspect of the proposed model involves 2 key aspects: collaboration between care robots and integration with medical equipment or systems. By connecting nursing robots with other types of care robots, a comprehensive and coordinated care environment can be established. Different care robots, such as those specializing in mobility assistance, those responsible for cognitive support, and those that provide social interaction, can work together to address the diverse needs of elderly people. For example, a mobility assistance robot can help an elderly person move around a house while a social interaction robot engages them in conversations and activities. This synergy enhances the overall care experience, ensuring that all aspects of an elderly people’s well-being are addressed efficiently and effectively. Integrating nursing robots with medical equipment and healthcare systems ensures seamless and real-time data sharing among the various devices and with healthcare providers. This includes linking robots with telehealth platforms, electronic health records (EHRs), and remote monitoring systems. For example, a nursing robot can collect health data from wearable devices and transmit them to the EHR, where healthcare providers can access and analyze them. Similarly, remote monitoring systems can alert robots to any immediate health concerns, prompting them to take appropriate actions or notify caregivers. By fostering networked collaboration both among care robots and between care robots and medical equipment and systems, more comprehensive and effective care can be delivered to the patient. This approach would not only improve the health monitoring and intervention capabilities of the robots but also facilitate a more holistic and integrated care model that meets the complex needs of the aging population.

### Intelligence-driven services

Intelligence-driven services leverage artificial intelligence, particularly machine learning, to enhance the capabilities of nursing robots. These technologies enable robots to learn from their interactions with the user, adapt to the user’s needs, and provide personalized care. For example, a primary health monitoring system consists of a sensor module, a data analysis module, and a visual interaction module. Through the aforementioned networked collaboration, various multimodal sensors can be used to collect environmental and physiological data of elderly patients. These sensors would perform local filtering on a collection board and transmit the data to a computer, where advanced techniques such as deep learning and big data-based algorithms can be to analyze this information. For example, computationally intensive convolutional neural networks with multiple hidden layers could be trained with high-performance computers, and the resulting models would be validated with data collected offline, enabling corresponding judgments and instructions for actuators [88]. Technologies such as the IoT and cloud computing allow robots to achieve autonomous decision-making, recognition, judgment, and reasoning and to automatically adjust their programs within a preset range on the basis of changes in external inputs. Flexible sensors can be integrated into clothing, mattresses, carpets, and other furniture in the living environment for data collection. For example, the reported Hadoop MapReduce technology enables parallel processing of large amounts of data collected from multiple wearable sensors placed on the subject’s left ankle, right arm, and chest. These data are transmitted through IoT devices to an integrated cloud and data analytics layer. Using a 3-tier architecture consisting of a perception layer, an integrated cloud layer, and a data analytics layer, it is possible to identify 12-lead records with an accuracy of 97.1% [89]. Furthermore, artificial intelligence algorithms can be used to analyze health data to predict potential health issues and recommend preventive measures. Machine learning can help robots improve their performance over time, becoming more effective in assisting with daily activities and providing emotional support.

### Precision service delivery

The goal of precision service is to utilize networked collaboration to create a massive user database containing individual health conditions and preferences that can then be leveraged to offer tailored care plans for the users. In particular, nursing robots can provide different services to elderly patients via speech recognition, facial expression recognition, gesture recognition, etc., including item delivery, dietary care, vital sign monitoring, rehabilitation training, personalized emotional communication, and companionship. Health monitoring robots could continuously monitor the users’ vital signs and activity data, transmitting this information to a comprehensive cloud-based network management platform. By applying deep learning techniques to the users’ health data, these robots can also develop personalized and holistic health profiles. If any issues are detected, alerts are sent to the users and healthcare providers via the network management platform. In terms of medication management, health management robots can utilize advanced machine learning algorithms to analyze patient data, optimize medication plans, and adjust dosages in real time on the basis of the patient’s current health status [90,91], thereby ensuring timely and accurate administration of medication. Rehabilitation training nursing robots can guide elderly users through personalized rehabilitation exercises, adjusting the intensity and type of exercise on the basis of real-time feedback from motion sensors and health data. These robots would use machine learning to track the user’s progress and modify the therapy programs to maximize physical recovery and health maintenance. Daily care robots could employ artificial intelligence to learn users’ preferences and routines, providing customized assistance that respects their daily habits and enhances their quality of life. Finally, emotional companionship robots could utilize natural language processing (NLP) and sentiment analysis to adapt their communication style on the basis of the users’ emotional responses, offering more naturalistic and supportive companionship. Artificial intelligence-driven analytics would enable all these robots to continually learn and adapt to the evolving needs of the users, ensuring that the provided care remains relevant and practical.

### Diversified integration

The networked intelligent elderly care model should incorporate a process of cross-disciplinary infiltration, embedding, and integrated development among different disciplines and industries. First, the model requires reaching across disciplinary barriers to facilitate robust cooperation between medical and engineering disciplines. From the perspective of industry, it should be acknowledged that technology plays a major role not only in elderly care but also in other fields such as real estate, transportation, and food. Intelligent devices and systems are essential elements of the living environment for elderly individuals. Thus, intelligence-based services are not only a solution to the problems in elderly care but also a natural result of industrial integration and development. Therefore, diversified integration reflects the sense of identity, convenience, specialization, and intelligence of the networked elderly care model.

### Standardized development

As nursing robots have become gradually adopted in different aspects of care, it has become necessary to establish standards for their functions such as patient monitoring, environmental evaluation, daily care, and rehabilitation. The designs of the user interfaces of and interaction with nursing robots are constantly iteratively optimized during the use of these devices, necessitating standardized requirements for the design of nursing robots. Indeed, a global consensus on standards for nursing robots still needs to be reached. Currently, in the absence of clear standards, a combination of subjective and objective indicators is leveraged to develop nursing robot operation standards from the perspectives of safety (mechanical, electrical, data, and biological safety), effectiveness (service, data, and operational effectiveness), and user friendliness (easy to understand, easily learning habits, easy to operate, and easily accepted). Technological progress and industrial development can be promoted through the use of standardized requirements for these systems.

## Implication of the Networked Intelligent Elderly Care Model

An example scenario implementing this networked, intelligent nursing robot-based model involves a semidisabled elderly male with right limb dysfunction who awakens to an alarm clock, at which point his bed automatically transforms into a wheelchair. He is then transported to the bathroom, where he relieves himself, and subsequently to the dining table. A nursing robot prepares the patient’s breakfast and assists with feeding while the elderly patient asks the robot to relay the weather and important news of the day. After the meal, the nursing robot retrieves any medication needed that day. By observing the individual’s limited mobility of the right limb and subdued mood, the nursing robot comforts him and encourages and guides him to complete the day’s rehabilitation exercises. Through real-time monitoring and cloud platform data processing, an increase in the patient’s blood pressure is detected, and an alarm prompt is issued. Community medical personnel immediately receive all vital sign monitoring information from the patient remotely and send a connection application. Physicians, rehabilitation therapists, and nurses can communicate with the elderly patient online about his medical conditions and rehabilitation plans.

## Challenges in Implementing the Networked Intelligent Elderly Care Model

Although the networked intelligent model could offer transformative changes to the care of elderly individuals, it is also associated with several controversies and potential risks. (a) Security: Nursing robots acquire substantial data during their service, increasing the potential consequences of privacy leaks. It is necessary to continuously improve existing data management and utilization systems to ensure the security of data collection and transmission [92]. (b) Autonomy: Through the machine learning algorithms used by nursing robots, the independent decision-making modes of the system are becoming increasingly more refined. However, there may be inconsistencies between the preferences of the patient and the autonomous decisions made by the robots [93]. We believe that the autonomy of the patient should be fully respected while guaranteeing their safety under robots. (c) Objectification and care: As the functions of nursing robots become increasingly sophisticated, they will be able to complete most care tasks but will not be capable of engaging in deep emotional communication the way humans can [94]. (d) Responsibility and attribution: Human nursing staff are responsible for all their care behaviors; however, nursing robots implement care following predetermined procedures or under remote human commands. When problems arise, individuals, including robot manufacturers, software designers, medical institutions, and operators, may be responsible.

The development of networked intelligent elderly care models is reliant on scientific and technological progress, which, in turn, requires interdisciplinary support and the widespread participation of experts in engineering, medicine, nursing, ethics, psychology, law, sociology, and other fields. Furthermore, refinements in relevant policies and laws are crucial; the responsible governments must improve nursing robot policies, laws, and regulations; clarify regulatory standards; and integrate the management fees, service fees, and nursing fees related to networked intelligent elderly care models with long-term care insurance to minimize the economic burden of elderly care.

## Conclusion

Nursing robots have demonstrated considerable capabilities in delivering intelligent monitoring, rehabilitation exercise, daily care, and companionship, all of which have the potential to assist in maintaining patient dignity during aging. Our proposed nursing robot-based networked intelligent model delivers elderly care through different approaches (networked collaboration, intelligence-driven services, precision service delivery, diversified integration, and standardized development) and will aid in the further “intelligentization” of nursing robots. By integrating nursing robots into the concept of active aging, the networked intelligent elderly care model promises to revolutionize the landscape of elderly care, making it more inclusive, effective, and aligned with the evolving needs of our aging population.
